# Heterogeneity, Measurement, and Clinical Implications of Oxygenation, Cell Signaling, and Redox Biology in Glioblastoma and Adult Diffuse Gliomas, with Context from Other Brain Tumors

**DOI:** 10.3390/antiox15040505

**Published:** 2026-04-19

**Authors:** Arabinda Das, Julian E. Bailes, Ann Barlow, Daniil P. Aksenov

**Affiliations:** 1 Endeavor Health Research Institute, Evanston, IL 60201, USA; 2 Pritzker School of Medicine, Chicago, IL 60637, USA; 3 Department of Neurosurgery, Endeavor Health, Evanston, IL 60201, USA; 4 Department of Radiology, Endeavor Health, Evanston, IL 60201, USA; 5 Department of Anesthesiology, Endeavor Health, Evanston, IL 60201, USA; 6 Department of Biomedical Engineering, Northwestern University, Evanston, IL 60208, USA; 7 Pritzker School of Medicine, University of Chicago, Chicago, IL 60637, USA

**Keywords:** glioblastoma, glioma, hypoxia, oxygenation, redox signaling, cell signaling, reactive oxygen species, ferroptosis, neurovascular uncoupling

## Abstract

Tumor oxygenation is a key determinant of cancer biology and treatment response, correlating with angiogenesis, recurrence, and malignant progression. Hypoxia is a defining feature of glioblastoma (GBM) and adult diffuse gliomas, generating low-oxygen niches that promote invasion, stem-like states, immune suppression, and resistance to radiotherapy and temozolomide, contributing to poor outcomes. Measuring tissue partial pressure of oxygen (pO_2_) and mapping its spatial heterogeneity can, therefore, inform mechanistic understanding and therapeutic development, including hypoxia-activated prodrugs, hypoxia-responsive gene therapy, and optimized radiotherapy planning. Although direct pO_2_ assessment is challenging, invasive probes and multimodal imaging can characterize regional hypoxia pre-operatively, support patient stratification, monitor treatment effects, and improve outcome prediction. This review summarizes oxygen dynamics in GBM; analyzes causes of hypoxia (rapid growth outpacing supply, diffusion-limited hypoxia, and abnormal/chaotic vasculature); compares methods to quantify oxygenation from direct measurements to noninvasive imaging surrogates; and evaluates preclinical and clinical strategies that target hypoxia to enhance standard therapy, including barriers to translation. We further integrate oxygenation with cell signaling and redox biology: oxygen gradients are transduced via hypoxia-inducible factor programs and redox-sensitive pathways (NRF2/KEAP1, NOX-derived ROS, nitric oxide/S-nitrosylation, and sulfur metabolic routes), shaping mesenchymal-like transitions and cell-death programs such as ferroptosis. Framing oxygenation as both a microenvironmental and redox-signaling variable positions oxygen imaging as an entry point to biomarker-guided therapies that exploit oxidative vulnerabilities.

## 1. Introduction

Gliomas, which develop from glial cells in the brain or spinal cord, are a major class of primary malignant tumors of the central nervous system (CNS) and are classified based on morphological and molecular characteristics [1,2]. Adult diffuse gliomas include isocitrate dehydrogenase (IDH)-mutant astrocytomas (grades 2–4), IDH-mutant oligodendrogliomas (grade 2 or 3) and glioblastoma (GBM) (IDH wild type, grade 4).

Accounting for more than half of astrocytomas and roughly one-fifth of primary malignant central nervous system tumors, glioblastoma (GBM) is the dominant malignant brain neoplasm in adults [3,4]. Epidemiologic estimates place its incidence at 3.19 per 100,000 individuals, and the median age at diagnosis is 64 years [5]. Management typically requires combined-modality care, including maximal safe resection, radiotherapy, systemic chemotherapy, selected targeted treatments, and supportive measures [3,4,5]. Even with this approach, durable survival remains uncommon, and the disease often causes progressive neurologic decline and marked loss of quality of life, affecting both patients and their families.

The imbalance between oxygen supply and consumption within the GBM tumor core often leads to hypoxia (insufficient oxygen), which is known to compromise the efficacy of radiotherapy and enhance aggressive GBM tumor behavior [6,7,8,9]. The clinically dominant consequence of hypoxia is local invasion and recurrence within the CNS rather than true extracranial metastasis, which remains rare [10]. Consequently, strategies to manipulate oxygenation levels within the GBM tumor to minimize hypoxia may have significant therapeutic potential.

Measurement of hypoxia may enable monitoring of treatment effects; for example, preclinical and clinical investigations have shown a substantial change in tumor oxygen—using partial pressure of oxygen (pO_2_) histographs and assays for hypoxia—after a single radiation fraction or during a course of fractionated radiotherapy [11,12,13,14,15,16,17]. While some useful information has been obtained using oxygen electrodes or fiber-optic probes to measure pO_2_ in human gliomas, use of such invasive methods for repeated measurements to assess pO_2_ changes over time within a tumor is not feasible [18,19,20]. However, noninvasive measurements of tumor tissue-level pO_2_ may have profound implications for our further understanding of GBM biology and the development of advanced cancer therapies. Here, and in the sections below, most noninvasive methods should be interpreted as tissue-level or surrogate oxygen measures rather than direct intracellular pO_2_ measurements [6].

**Figure 1:**
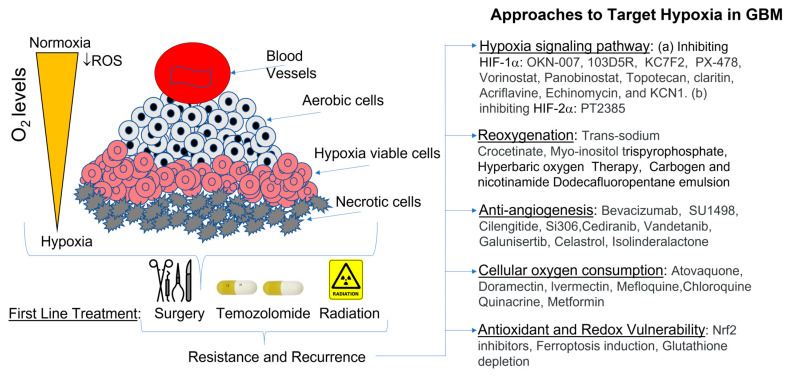
(**Left**) Conceptual overview of oxygen gradients in glioblastoma. The schematic illustrates the transition from relatively normoxic, vascularized regions to hypoxic, viable zones and necrotic areas. (**Right**) Representative oxygen- and hypoxia-targeted therapeutic strategies discussed in this review, including inhibition of hypoxia-signaling, reoxygenation, anti-angiogenic normalization, modulation of oxygen consumption, and redox-based vulnerabilities. ROS: reactive oxygen species; HIF-1α: hypoxia-inducible factor 1α; OKN-007: Oklahoma nitrone 007; 103D5R, PX-478. KC7F2, KCN1: experimental HIF-1 inhibitors; HIF-2α: hypoxia-inducible factor 2α; PT2385: experimental HIF-2 inhibitor; SU1498: experimental vascular endothelial growth factor receptor 2 (VEGFR2) inhibitor; Nrf2: Nuclear factor erythroid 2-related factor 2.

In this literature review, we discuss oxygen dynamics in GBM—characterized by significant but spatially and temporally heterogenous hypoxia within the tumor—which affects tumor progression and treatment resistance. We describe how the rapid growth and chaotic tumor-associated vasculature of GBM leads to diffusion-limited hypoxia and discuss methods of measuring oxygenation in brain tumors. Linking oxygenation to tumor biology—molecular signatures, cell states, and immune microenvironment—we discuss the clinical implications of oxygen-targeted therapeutic strategies in neuro-oncology (Figure 1). Although examples from adult diffuse gliomas, metastases, meningiomas, and pediatric tumors are included, the most mature human oxygenation literature still centers on GBM and adult diffuse gliomas [21,22,23].

Importantly, hypoxia in GBM should not be viewed solely as an oxygen deficit. Changes in oxygen tension alter redox signaling by modulating mitochondrial and NADPH oxidase-derived reactive oxygen species (ROS), nitric oxide bioavailability, and the antioxidant systems that maintain intracellular redox balance [24]. This redox layer connects oxygenation status to cell state transitions, immune suppression, and therapeutic resistance, and provides a mechanistic bridge between imaging-defined hypoxia and actionable biology [25,26].

At the molecular level, oxygen tension converges with NRF2/KEAP1, PI3K/AKT/mTOR, Wnt/beta-catenin, Notch, and STAT3 signaling, especially in tumor stem-like cells, such that redox homeostasis and cell state plasticity should be considered together rather than in separate conceptual boxes [25,27,28].

### Novelty Statement

Several important reviews have addressed adjacent components of this field, but the literature remains compartmentalized into three largely separate streams: oxygenation and hypoxia-imaging reviews focused on measurement, modality performance, and treatment adaptation [23,29,30,31,32]; hypoxia- and hypoxia-inducible factor (HIF)-centered glioblastoma reviews focused on microenvironmental biology, invasion, immunity, and treatment resistance [7,8,33,34,35,36,37,38,39]; and redox and oxidative-stress reviews centered on ROS, antioxidant buffering, NRF2, redox-dependent resistance, and stem-like states [27,40,41,42,43,44,45,46,47]. To our knowledge, no prior review has integrated these domains within a single brain tumor-focused framework that moves explicitly from oxygen measurement to redox mechanisms to therapeutic application. The present review is therefore distinct in four ways: it clarifies what invasive probes and multimodal imaging methods actually measure and how those readouts should be interpreted biologically; it links oxygen gradients to redox-sensitive signaling networks including HIFs, NRF2/KEAP1, NOX-derived reactive oxygen species (ROS), nitric oxide/S-nitrosylation, glutathione-thioredoxin buffering, sulfur metabolism, and ferroptosis; it embeds these pathways in glioma-specific processes such as neurovascular uncoupling, peritumoral biology, stemness, mesenchymal-like transition, immune suppression, and treatment resistance; and it reframes oxygen imaging not as a descriptive endpoint alone but as an entry point for biomarker-guided, redox-aware therapeutic stratification in neuro-oncology. In this sense, the manuscript occupies a genuine and timely niche at the intersection of tumor oxygenation, cell signaling, and redox biology in gliomas and related brain tumors.

## 2. Oxygenation in Brain Tumors: Concepts and Brain-Specific Constraints

Although the brain comprises only 2% of the body’s weight, it consumes 20% of the body’s oxygen [37]. Neurons have a high baseline metabolic demand required to maintain essential functions such as electrical signaling and may die within minutes without sufficient oxygen. Rapidly proliferating tumor cells must compete with the surrounding functional brain tissue for oxygen supply, thus GBM tumors are typically hypoxic. Microscopic analyses of GBM reveal multiple hypoxic regions, and GBM tumor oxygenation is severely compromised and highly heterogeneous compared to normal tissue. The median pO_2_ in GBM tumors is 10 mm Hg, and approximately 25% of the tumor is hypoxic (pO_2_ ≤ 2.5 mm Hg) [22]. Studies in GBM animal models showed that certain gliomas (C6, F98, and U251) are also hypoxic, with pO_2_ in the range of 7–12 mmHg, while others (9 L) are well-oxygenated [11,48]. Whereas normal tissue oxygenation is independent of hemoglobin level over the range of 8–15 g/dL, hypoxia is more pronounced in anemic patients and even in some cancer patients with normal hemoglobin levels [16,49,50,51]. Direct measurements also show lower oxygen tension in human glioma and, in some cases, in adjacent peritumoral tissue [16,21,50]. Throughout this review, oxygenation refers primarily to the balance between oxygen delivery and oxygen consumption at the tissue level, often expressed as pO_2_, rather than to any single imaging-derived signal [16,49,50].

Tumor hypoxia is caused by (i) high oxygen consumption due to rapid cellular proliferation, (ii) inadequate tissue perfusion due to disorganized tumor neovascularization [52,53,54,55,56,57], and (iii) blood vessel occlusion, edema, and tissue necrosis, all of which limit oxygen availability within the tumor.

The rapid proliferation of tumor cells quickly outpaces the growth of new, functional blood vessels, meaning the center of the tumor is located far from its blood supply. Tumor-induced blood vessels are often structurally and functionally abnormal and leaky, leading to chaotic blood flow and impaired oxygen delivery. As a result of the aberrant, dysfunctional tumor vasculature and increased diffusion distance, oxygenation levels fail to meet the high metabolic demands of the rapidly growing tumor [52,58,59,60,61].

Tumor growth, and the associated development of edema, within the confines of the rigid skull, increases intracranial pressure (ICP), which can compress blood vessels and further restrict blood flow and oxygen delivery. This, in turn, can cause or worsen cellular injury and edema, creating a vicious cycle. The increased pressure from the tumor and surrounding edema can also compress the fragile capillaries that supply healthy surrounding tissue; thus, reduced blood flow causes hypoxia in both the tumor and normal brain tissue, and hypoxic regions are also found in the peritumoral brain tissue. This combination of rising ICP and local hypoxia can impair or block the function of nearby healthy neurons [62]. While tumor location may compromise specific brain functions—for example, a tumor near the motor or visual cortex can cause motor or vision deficits, respectively [63]—increased competition for glucose and oxygen can result in a widespread depression of metabolism in the cerebral cortex, potentially resulting in encephalopathy [64].

Low pO_2_ is a critical factor driving aggressive tumor growth, treatment resistance, and poor patient prognosis. There is a significant relationship between tumor hypoxia and histological grade, with lower median pO_2_ values in high-grade than in low-grade gliomas [16,21,22]. When oxygen levels drop, hypoxia-inducible factors (HIFs) are stabilized and activated. HIFs are transcription factors that are the primary regulators of adaptive responses to oxygen deficiency in tumor cells [38]. High levels of HIFs and other pro-angiogenic factors, such as vascular endothelial growth factor (VEGF), drive the formation of new blood vessels, a process known as angiogenesis. HIFs also influence immune cell behavior by promoting VEGF-driven vascular remodeling, myeloid cell recruitment/polarization, and immune-checkpoint signaling thereby promoting an immunosuppressive microenvironment [7,37,65]. Hypoxic conditions drive cancer cells to switch their metabolism from efficient oxidative phosphorylation to less efficient anaerobic glycolysis, which also contributes to an acidic extracellular environment. These effects are independent of tumor characteristics (e.g., size, stage, histology, and grade) and patient demographics.

Another brain-tumor-specific point is that oxygen deprivation is often intermittent rather than static. Cycling hypoxia and reoxygenation generate bursts of mitochondrial and NOX-derived ROS that amplify HIF signaling, angiogenesis, invasion, and radioresistance. In GBM, NOX4-dependent ROS signaling under cycling hypoxia has been linked experimentally to tumor progression and treatment resistance, reinforcing the idea that oxygen fluctuation is itself a biologic signal [66,67,68].

## 3. Microvasculature, Perfusion, and Neurovascular Coupling/Uncoupling as Determinants of Oxygenation

GBM exhibits abnormal vascular morphology—characterized by disorganized, tortuous, blood vessels—which disrupts perfusion. The disordered vascular system in and around tumors occurs because GBM cells can both grow around existing blood vessels (vascular co-option) and induce new vessels to grow (angiogenesis). Most blood vessels associated with angiogenesis in aggressive GBM are immature and dysfunctional, lacking proper pericyte and endothelial cell coverage, resulting in a leaky vascular network that is not subject to normal regulatory mechanisms [69,70]. These abnormal vessels cannot maintain a consistent, efficient blood supply; blood flow is highly heterogeneous, with some areas experiencing high flow and others being poorly perfused or experiencing stasis. GBM progression is also linked to the formation of large-caliber vessels, due to excessive angiogenesis [71], that can become twisted and convoluted, which can both increase blood volume and compromise flow. Greater vessel caliber can increase local blood volume, but convoluted or inefficient flow paths do not guarantee effective perfusion or oxygen delivery [69,70,71].

The abnormal vessels allow plasma and other substances to escape into the surrounding brain tissue, which can contribute to edema. These vascular abnormalities also limit drug delivery to the tumor; systemic drugs cannot adequately reach poorly perfused regions or are quickly lost from leaky vessels. The poor accumulation of conventional chemotherapy drugs in tumors is a direct result of this inefficient vasculature—and is a major factor undermining the effectiveness of these therapies.

In a healthy brain, cerebral autoregulation keeps a steady blood supply by adjusting blood flow in response to pressure changes, while neurovascular coupling (NVC) functions as an efficient, real-time delivery system, precisely linking local blood flow to neural activity to ensure adequate oxygen and glucose delivery. A rise in neuronal activity is directly followed by an increase in local cerebral blood flow [72,73], ensuring that active brain regions receive the necessary oxygen and glucose. Normal brain function relies on the ability to maintain constant blood flow despite fluctuations in blood pressure (cerebral perfusion pressure, CPP). These mechanisms are disrupted by the abnormal tumor-associated vascular networks, leading to impaired autoregulation and neurovascular ‘uncoupling’ [74], such that the ability to adapt blood flow to pressure changes or in response to neural activity in the surrounding healthy brain tissue is distorted. The failure to maintain consistent blood flow can have functional consequences, leading to an inadequate supply of oxygen for active neurons in some areas, while other areas may receive too much blood, increasing the risk of brain damage [75]. Heterogeneous blood flow within different regions of the tumor also complicates uniform treatment of the tumor. In gliomas, invading tumor cells can disrupt astrocyte–vascular signaling and extend neurovascular uncoupling into the peritumoral region, which may produce blunted or false-negative fMRI activation maps unless the physiologic context is considered [69,73].

In the normal cerebral vasculature, homeostatic mechanisms also regulate blood flow in response to changes in CO_2_/O_2_ levels. Cerebral blood flow increases in response to rising CO_2_ levels (hypercapnia) and decreases during falling CO_2_ (hypocapnia) via vasodilation and vasoconstriction, respectively [76]. In local healthy brain tissue, vascular beds dilate in response to neuronal activation [77,78,79]. In addition to the disruption in cerebral autoregulation and NVC [80,81], GBM tissue demonstrates diminished CO_2_/O_2_ reactivity compared to healthy tissue, which may be due to the inefficient, immature neovasculature of GBM tumors or to the effects of tissue hypoxia. Impaired CO_2_/O_2_ reactivity on Blood oxygen level-dependent (BOLD, see Section 4.2.1) or related MRI therefore reflects altered vascular responsiveness and neurovascular uncoupling as much as altered oxygen delivery, and should not be interpreted as a direct readout of tissue pO_2_ [55,73,74,80,81,82].

These vascular abnormalities are also redox-active. Altered nitric oxide (NO) bioavailability, ROS scavenging of NO, and thiol-based post-translational modifications can reshape vascular tone, endothelial permeability, and barrier stability, meaning that the glioblastoma vascular phenotype reflects disordered signaling chemistry as well as disordered anatomy. This is exemplified by S-nitrosylation-dependent endothelial dysfunction in GBM models [26,83].

## 4. Methods to Measure Oxygenation in Brain Tumors: From Invasive Probes to Multimodal Imaging

Detection of tumor hypoxia in the clinical setting may allow assessment of the tumor’s potential to develop an aggressive phenotype or treatment resistance, both of which lead to a poor prognosis. Tools to characterize tumor oxygenation status are essential for the therapeutic management of GBM, in order to refine prognosis, assess and adapt the treatment regimen for optimal therapeutic efficacy, and potentially to identify high-risk patients for individualized and/or more intensive treatment schedules (Table 1).

Methods to measure brain tumor oxygenation range from invasive, direct measurement with oxygen electrodes and optical fibers to non-invasive, multimodal imaging techniques such as functional MRI (fMRI) and positron emission tomography (PET) scans [18,84,85,86]. Invasive methods offer direct, real-time measurements and can show dynamic changes in real-time, but provide only focal data, have poor sensitivity at high oxygen levels, and are difficult to use in clinical settings. Non-invasive methods (e.g., using BOLD fMRI and PET to visualize and quantify oxygen related changes) allow for whole-tumor assessment [87]. In routine GBM care, contrast-enhanced anatomical MRI remains the standard pre- and early post-operative study, whereas diffusion/perfusion MRI and, in selected centers, amino-acid PET are used as adjuncts for tumor delineation, recurrence assessment, and treatment planning rather than as direct oxygen measurements [18,23,84,85,86,87]. However, all methods have pros and cons, and no single method meets all criteria for an ideal technique to assess local oxygenation; the optimal method depends on the specific clinical or research goal. For clarity, the techniques described below are not interchangeable: electrodes and EPR estimate local tissue pO_2_, BOLD primarily reflects deoxyhemoglobin-sensitive signal [88], perfusion MRI reflects blood delivery, and nitroimidazole PET reflects tracer retention in hypoxic tissue [6,89,90] (Table 2).

For a redox-focused interpretation, it is important to distinguish oxygen assessment from redox assessment. Oxygen imaging can identify low-oxygen habitats, vascular responsiveness, or tracer-retaining hypoxic tissue, but it does not directly quantify ROS flux, glutathione/thioredoxin buffering, nitric oxide signaling, or ferroptosis susceptibility. Measurement of these downstream properties likely requires integration with radiogenomics, tissue profiling, or metabolic biomarkers [6,9,91].

### 4.1. Invasive Methods

#### 4.1.1. Polarographic Oxygen Electrodes

Inserted directly into tissue, these small metal probes comprise a fine needle electrode with a platinum cathode and a silver/silver chloride anode covered by an oxygen-permeable membrane. Based on the Clark electrode principle, polarographic electrodes measure the electrochemical reduction of oxygen at the cathode; the resulting electric current is proportional to the local pO_2_. This approach has the advantage of providing direct real-time, quantitative point measurements of pO_2,_ and is often considered the gold standard [21], but has the significant disadvantages of being invasive and measuring only a focal area (50–100 cells), thus offering a limited view of the tumor. Measurements are also highly dependent on the location of the probe tip, which can be challenging to precisely control, and making repeated measurements at the same location is impractical as frequent insertion of the probe can cause local trauma, hemorrhage, and blood flow artifacts, which can influence pO_2_ readings.

#### 4.1.2. Fiber Optic Probes

This technique uses a luminophore that fluoresces in response to O_2_ levels, allowing sensitive detection of low O_2_. However, like polarographic electrodes, this technique is invasive and limited to focal measurements.

#### 4.1.3. Electron Paramagnetic Resonance (EPR) Oximetry

This magnetic resonance imaging (MRI)-based technique uses tiny, implanted oxygen-sensitive paramagnetic crystals such as lithium phthalocyanine (LiPc) or lithium octa-n-butoxynaphthalocyanine (LiNc-BuO) to measure pO_2._ These probes are introduced as small particulate deposits through a minimally invasive needle, after which repeated measurements can be obtained from the same site [92,93]. Their interaction with the natural paramagnetic properties of molecular oxygen [12,19] causes a broadening of the EPR spectrum that is linearly proportional to the local pO_2_. Later measurements can be performed non-invasively by applying a magnetic field, allowing for continuous or repeated quantitative assessment of changes in pO_2_ over days or weeks at the same site—a capability not possible with polarographic or fiberoptic probes. The particulate probes are metabolically inert and do not consume oxygen; thus, the measurements do not alter local oxygen levels. Advanced multi-site EPR oximetry can measure pO_2_ simultaneously at multiple locations within a tumor and the contralateral brain, offering crucial, spatially specific information on pO_2_ heterogeneity within the tissue.

### 4.2. Non-Invasive (Imaging) Methods

Diagnostic tools such as perfusion-weighted MRI are currently used to assess blood flow heterogeneity, which can help in grading tumors, planning treatment, and monitoring responses [18,53,80,82,87,94].

#### 4.2.1. BOLD/T2* MRI

Blood oxygen level-dependent (BOLD) functional MRI (fMRI) is based on the different magnetic properties of oxygenated and deoxygenated hemoglobin [80,81,89]. It measures signal changes, often using the T2* relaxation parameter, to create maps of relative blood oxygenation. BOLD MRI is noninvasive and widely available, with high spatiotemporal resolution. It uses a standard MRI technique that does not require exogenous contrast agents or radioactive tracers and can capture rapid changes in oxygenation. BOLD fMRI is dependent on a complex interplay of blood flow, blood volume, and oxygen consumption [81,95]. As the tumor and associated edema affect these parameters, a decreased BOLD signal—reflecting neurovascular uncoupling, a delayed hemodynamic response, and signal heterogeneity and asynchrony—allows for differentiation of tumor tissue from normal brain. Reduced CO_2_ reactivity, measured with BOLD-fMRI, can extend beyond the visible tumor margin and correlate with the size of the surrounding edema.

A major limitation is that BOLD provides a relative measure of oxygenation, not a quantitative one. Hypoxic tissue may show a minimal change in BOLD signal in response to extra oxygen because it is consumed immediately, unlike in healthy tissue. The signal can also be affected by factors other than oxygenation, such as hydration status and susceptibility artifacts. The signal can also be dominated by large draining veins that may obscure local tissue oxygenation.

#### 4.2.2. Quantitative BOLD (qBOLD)

This technique uses a more sophisticated model to separate the various components contributing to the BOLD signal, allowing for noninvasive quantitative mapping of the oxygen extraction fraction (OEF) and deoxygenated blood volume (DBV), and providing a noninvasive approach to assess the proportion of oxygen removed from the blood by the tissue. By combining OEF with blood flow data, qBOLD can estimate the cerebral metabolic rate of oxygen (CMRO_2_). Major limitations are that the technique relies on a complex biophysical model, and simplifying assumptions can affect accuracy [96,97,98]. Quantifying OEF is challenging, and separating the various components that contribute to the signal can be difficult and prone to error.

#### 4.2.3. Perfusion MRI

This technique measures blood flow to tissues by tracking the passage of a tracer, which can be an injected contrast agent like gadolinium or magnetically labeled water (in arterial spin labeling, or ASL) [52,54,55,56,57]. Changes in perfusion can be used to infer tissue oxygenation. This method has high enough sensitivity to visualize the microvasculature, which effectively quantifies blood delivery to tissue. It has high spatial resolution and offers excellent anatomical detail. However, it has a few limitations in that it measures blood flow, not the actual oxygen levels in the tissue—and hypoxia can exist even with adequate blood flow—and contrast agents take time to clear from the body, limiting the ability to repeat measurements over a short time period.

#### 4.2.4. Oxygen-Enhanced MRI (OE-MRI)

The change in the magnetic properties of a tissue (specifically, the longitudinal relaxation rate) when a patient breathes 100% oxygen [99,100,101] indicates the responsiveness of the tissue to an increased oxygen supply. This technique can directly assess the capacity of a tissue to accept and use oxygen, rather than just measure baseline oxygen levels, and it can track changes in oxygenation over time, which is useful for monitoring treatment response. However, it has some limitations, as it provides a relative or semiquantitative measure of oxygenation and cannot calculate absolute pO_2_ values, requires careful experimental control, and the signal can have a low signal-to-noise ratio in some areas—for example, in the lung. OE-MRI therefore reflects responsiveness to increased inspired oxygen and should not be interpreted as a universal absolute pO_2_ map [99,100,101].

#### 4.2.5. Nitroimidazole Positron Emission Tomography (PET) Tracers

Radioactive tracers that target hypoxia create images of oxygen-deprived areas in PET scans, providing a specific, quantitative map of tissue regions with very low oxygen tension, regardless of blood flow, with a high molecular specificity for hypoxic tissue. FMISO (^18^F Fluoromisonidazole), the most extensively studied hypoxia tracer in humans and animals [82], and [64Cu]-Diacetyl-bis(N4-methylthiosemicarbazone) ([64Cu]-ATSM) accumulate specifically in hypoxic cells. Increased uptake is associated with higher-grade gliomas and poor prognosis. This approach has the advantage of showing the extent and variability of hypoxia throughout the tumor volume, but requires the use of ionizing radiation in the tracer, limiting repeated use in the same patient, and does not provide absolute values, relying on thresholds or kinetic modeling for quantification. Tracers like FMISO accumulate slowly in hypoxic areas, requiring several hours between injection and imaging, and PET imaging has lower spatial resolution than MRI. As hypoxia is dynamic, repeat FMISO scans may be necessary to show variations in hypoxia over time. Note that FMISO and related nitroimidazole PET scans show tracer retention in hypoxic tissue, which is not numerically identical to a direct pO_2_ measurement [22,39,84,85,90].

#### 4.2.6. Combined PET–MRI

This hybrid technology combines the strengths of both PET and MRI into a single scan [102,103], providing a powerful combination of highly specific metabolic data (from PET) and excellent anatomical and functional detail (from MRI). Simultaneous data acquisition provides better alignment of PET and MRI data than fusing separately acquired images and may decrease overall radiation dose compared to a combined PET/CT scan. However, combined PET/MRI scanners are expensive and not as widely available as PET/CT or standalone MRI systems [104], and PET/MRI scanning protocols can be slower than PET/CT. The complex nature of the combined hardware may create technical hurdles due to potential interference between the two modalities.

Other methods, such as functional near-infrared spectroscopy (fNIRS) and photoacoustic imaging (PAI) have been used to measure oxygenation [105,106,107,108,109,110,111]. fNIRS, often used, for example, in radiotherapy treatments, measures changes in hemoglobin oxygenation levels, while PAI uses light pulses to generate sound waves, which can be used to check changes in oxygen saturation, particularly in response to therapy. Dynamic susceptibility contrast (DSC) and dynamic contrast enhanced (DCE) MRI are perfusion techniques that can provide information related to blood flow and vascular permeability, which are indirect indicators of hypoxia.

Radiological imaging offers non-invasive methods to map and quantify hypoxia in gliomas, providing spatial information that complements genetic data [23,112,113,114,115]. Radiomics uses artificial intelligence (AI) to extract quantitative features from standard CT, PET, and MRI sequences (T1, T2, FLAIR) to serve as predictive markers for hypoxia [116]. A recent systematic review also emphasized that MRI-based hypoxia surrogates in brain malignancy remain promising but are only partially validated against direct oxygen measurements or PET, so modality-specific limitations should be explicitly recognized when interpreting oxygenation maps [23].

## 5. Spatial and Temporal Heterogeneity of Oxygenation in Different Brain Tumor Entities

Spatial and temporal oxygenation heterogeneity in brain tumors [35,117,118,119,120] is a major challenge for treatment, because areas of hypoxia can promote therapeutic resistance and lead to tumor recurrence.

Different tumor entities, such as GBM and brain metastases, show distinct oxygenation patterns and metabolic microenvironments. Intratumoral oxygenation heterogeneity is a defining feature of aggressive cancers like GBM, in which different subregions of a tumor show distinct biological behaviors due to varying microenvironmental pressures, including variation in perfusion, oxygenation, and cellular composition across the tumor core, margins, and surrounding brain tissue. Aggressive tumors show heterogeneous blood flow, leading to both highly vascularized and severely under-perfused areas. In GBM, vascular-rich perivascular niches are often associated with tumor stem cells and higher cellularity. However, an increase in vessel density does not always guarantee good perfusion, as abnormal, dysfunctional vessels can increase hypoxia. Poorly perfused areas result in hypoxic conditions that drive metabolic heterogeneity by forcing cancer cells to switch from oxygen-dependent metabolism to glycolysis for survival, producing an acidic microenvironment (acidosis) which promotes a more aggressive, therapy-resistant, and invasive phenotype. A hypoxic environment drives tumor cells to adapt and evolve, promoting cancer cell clones that are better adapted to survive and proliferate in a low-oxygen environment, and that are also more aggressive, treatment resistant, and able to evade the immune system [35,117,118,119,120]. Hypoxia thus promotes further tumor growth, aggressiveness, and local invasion and recurrence within the CNS, triggering a cascade of events that drives progression. In GBM, hypoxia can lead to the formation of pseudopalisading necrosis, a hallmark of high-grade disease, where tumor cells surround blocked vessels.

### 5.1. Redox Heterogeneity Parallels Oxygenation Heterogeneity

Oxygenation heterogeneity is accompanied by redox heterogeneity. Distinct regions within the same glioblastoma can differ in antioxidant reserve, cysteine availability, lipid peroxidation, and ferroptosis sensitivity, meaning that areas with similar oxygenation may not share the same oxidative vulnerability. At the invasive edge, GBM cells show increased levels of oxidative stress-associated metabolites and targetable transsulfuration pathway activity, while antioxidant network signatures cluster GBM into distinct redox-resistant phenotypes [91,121,122].

### 5.2. Identifying Heterogeneity in Tumors

Heterogeneity is studied in several ways. Advanced neuroimaging techniques like PET and MRI are used to create “habitat maps” of the tumor, showing the spatial relationship between hypoxia, perfusion, and other features. Perfusion MRI and PET imaging can reveal variations in blood flow and oxygen metabolism, with poorly perfused subregions often correlating with worse patient outcomes. Oxygen-enhanced MRI (OE-MRI) and BOLD fMRI can non-invasively track changes in tumor oxygenation and perfusion. Invasive, oxygen-sensing electrodes that provide a direct measure of oxygen tension have demonstrated pervasive fluctuation in oxygenation levels within tumors. Combining these techniques allows researchers to map hypoxic areas, new blood vessel formation (neoangiogenesis), and altered tissue structure, helping to better define tumor habitats.

### 5.3. Oxygenation Heterogeneity in Infiltrative Margins, Edema, and Peritumoral Cortex

A major challenge in treating gliomas is that tumor cells infiltrate the surrounding normal brain tissue well beyond what is visible with standard imaging techniques. These infiltrating cells at the tumor periphery are a primary cause of recurrence. The peritumoral brain zone (PBZ)—the area of brain tissue surrounding the tumor—often shows edema visible as T2/FLAIR hyperintensity on MRI. Evidence indicates that this normal-appearing region is far from homogeneous and includes scattered tumor cells. Radiomics analysis, which uses artificial intelligence and machine learning techniques to extract large amounts of data from medical images, has revealed that even within this peri-edema region, there is significant heterogeneity. Features extracted from the peri-edema can predict the location of future tumor recurrence. Regions close to the tumor core with those farther away show different radiomic signatures that correlate with the degree of infiltration. The invasive tumor cells found in the margins and peritumoral edema have different molecular and genomic features than those in the core of the tumor. Recent research using multiomic techniques reveals distinct genetic signatures within these infiltrative niches that explain their therapy resistance. Direct polarographic studies are important here because regions below 2.5 mmHg have been measured not only in glioma but also in the surrounding peritumoral brain, supporting the view that the PBZ is biologically active rather than purely vasogenic edema [21]. These infiltrative and peri-edema regions therefore matter not only for invasion biology but also for oxygen mapping, because clinically relevant hypoxic foci can extend beyond contrast-enhancing tumor [21,93].

### 5.4. Oxygenation Heterogeneity in High- and Low-Grade Gliomas, Metastases, Meningiomas, and Pediatric Tumors

In general, high-grade gliomas and metastases are characterized by significant spatial and temporal oxygenation heterogeneity, with high levels of hypoxia [123,124,125,126,127,128]. In contrast, low-grade gliomas and meningiomas tend to have less severe hypoxia, though heterogeneity still exists, especially in higher-grade subsets of these tumors. Data on pediatric tumors are lacking, but studies also point to oxygenation heterogeneity that can be therapeutically targeted.

#### 5.4.1. High-Grade Gliomas (HGGs)

HGGs, such as glioblastoma (GBM), feature highly hypoxic core regions that are resistant to treatment, while the peripheral regions are often more oxygenated. These differences are driven by the disorganized, dysfunctional tumor vasculature that cannot keep up with the oxygen needs of rapidly dividing tumor cells. Hypoxia-avid tracers show high uptake in the hypoxic regions of HGGs. Oxygen levels in HGGs fluctuate over time, with frequent transient periods of hypoxia and reoxygenation, likely due to unstable blood flow. These dynamic shifts are linked to more aggressive tumor behavior and resistance to radiation and chemotherapy. In patients with GBM, higher pretreatment FMISO-defined hypoxic burden has been associated with shorter time to progression and poorer survival, indicating that hypoxia burden is clinically meaningful rather than purely descriptive [22].

#### 5.4.2. Low-Grade Gliomas (LGGs)

LGGs are less commonly associated with the severe hypoxia seen in HGGs, but they are not uniformly oxygenated. PET and MRI demonstrate that the spatial distributions and patterns of heterogeneity in LGGs differ from those in HGGs. Hypoxia may be involved in the progression of LGGs to more aggressive forms, and imaging findings suggest a correlation between tumor location, oxygenation, and malignancy. While less studied than in HGGs, temporal changes in oxygenation contribute to the evolution of LGGs, and the development of more aggressive, treatment-resistant clones is linked to hypoxic conditions over time. Quantitative FMISO-derived ptO_2_ mapping likewise supports lower oxygen tension in glioblastoma than in less aggressive gliomas [85].

#### 5.4.3. Metastases

Brain metastases often feature marked spatial oxygenation heterogeneity. Their vascularity and resulting oxygenation profiles vary significantly depending on the primary tumor type and specific microenvironment. As with gliomas, tumor oxygenation in metastases can fluctuate dynamically. This has therapeutic implications, as modulating oxygen levels may enhance the efficacy of radiation or chemotherapy in selected settings. Imaging techniques such as OE-MRI and other hyperoxygenation approaches have been explored in brain metastases, suggesting that oxygenation can be modulated in at least some lesions.

#### 5.4.4. Meningiomas

High-grade meningiomas also show significant intratumoral heterogeneity, including variations in apparent diffusion coefficient (ADC) on MRI, which can correlate with cellular proliferation. Oxygenation heterogeneity, while not as prominent as in HGGs, does exist and is therapeutically relevant, especially for radiation treatment planning. Meningiomas respond heterogeneously to oxygen-enriched breathing gas mixtures, with variations in oxygenation effects and vascular changes. This indicates that oxygenation is not static and can be manipulated, which is important for optimizing radiosensitivity. BOLD MRI can be used to measure changes in blood oxygenation in meningiomas in response to respiratory challenges (e.g., hyperoxic-hypercapnic gas).

From a redox and cell-signaling perspective, meningiomas are increasingly relevant rather than peripheral to this discussion. Recent work in WHO grade 2/3 meningiomas identified oxidative stress-based molecular subtypes with distinct immune-checkpoint profiles and drug sensitivities, suggesting that oxidative state may be used to stratify aggressive meningiomas beyond conventional grading alone [129]. In parallel, meningioma signaling studies indicate that mTOR activity is linked to autophagy and redox homeostasis, while ferroptosis sensitivity can be shaped by NF2/E-cadherin/MEF2C signaling and by NOTCH3-driven fatty acid oxidation, placing oxidative vulnerability at the intersection of lineage-defining signaling and therapy resistance [130,131,132].

#### 5.4.5. Pediatric Tumors

Data on oxygenation heterogeneity in pediatric brain tumors, such as medulloblastoma and pediatric gliomas, remain limited. Imaging studies support biologic heterogeneity among pediatric brain tumors, but dedicated oxygenation-focused data are far fewer than in adult diffuse glioma [86,126]. Hypoxia is nevertheless likely to influence aggressiveness and treatment response in selected pediatric CNS tumors, but the current evidence base is insufficient to generalize adult GBM paradigms directly to pediatric disease.

### 5.5. Oxygenation Effects on Responses to Treatment

#### 5.5.1. Radiotherapy

Radiotherapy primarily kills cancer cells by causing DNA damage via ROS. Since oxygen is necessary for this process, hypoxic regions of a tumor are more resistant to radiation [133]. After radiotherapy, tumor oxygenation can change due to two main phenomena. As radiation kills the well-oxygenated cancer cells, the overall oxygen consumption within the tumor decreases. This improves the availability of oxygen for previously hypoxic areas. In addition, as the tumor shrinks, the distance oxygen must travel from blood vessels to cancer cells is reduced, improving oxygen diffusion. In some cases, radiotherapy can temporarily “normalize” the tumor vasculature by damaging immature endothelial cells, leading to more stable, functional blood vessels and improved blood flow. These changes may enable targeting of cancer cells that were initially resistant to radiotherapy. However, despite the general trend of reoxygenation, some forms of radiotherapy can induce or increase tumor hypoxia. Certain high-dose-per-fraction radiation protocols, such as stereotactic body radiotherapy (SBRT), can damage the tumor vasculature, leading to an initial increase in hypoxia. This can trigger survival mechanisms and potentially hinder the anti-tumor immune response. High radiation doses can damage blood vessel endothelial cells, leading to cell death and blockage of oxygen supply to cancer cells. Even after reoxygenation occurs, the oxidative stress induced by radiation can paradoxically stabilize HIF-1, maintaining radioresistance through hypoxia-related signaling. This oxygen-dependent fixation of radiation-induced damage is the biologic basis for the classic oxygen enhancement effect [32,133].

Importantly, radiation response is governed not only by oxygen fixation chemistry at the moment of treatment but also by how surviving glioma cells restore antioxidant buffering after irradiation. Reoxygenation and redox rewiring therefore occur in parallel during treatment adaptation [25,47].

#### 5.5.2. Anti-Angiogenic Therapy

Anti-angiogenic therapy is designed to inhibit the formation of new blood vessels, with the goal of “starving” the tumor of oxygen and nutrients [134]. The effect on tumor oxygenation, however, is complex and often transient. Some anti-angiogenic agents, particularly when administered at low doses or intermittently, can prune immature and leaky blood vessels while allowing the more mature vessels to function effectively. This transient period, known as the “normalization window,” can improve blood flow and oxygenation, making the tumor more responsive to treatments such as radiotherapy. However, other anti-angiogenic approaches may destroy tumor blood vessels, leading to a significant decrease in blood flow and an increase in tumor hypoxia, with consequent increases in tumor aggressiveness and treatment resistance due to selection for hypoxia-adapted clones. Compensatory mechanisms, including co-option of existing vessels or activation of alternative angiogenic pathways, can lead to resistance against anti-angiogenic drugs over time.

### 5.6. Clinical Implications of Intratumoral Heterogeneity

Understanding intratumoral heterogeneity is critical for improving cancer treatment and overcoming therapy resistance, as different tumor regions respond differently to therapy. For example, hypoxic regions are resistant to both chemotherapy and radiation. Identifying invasive, treatment-resistant regions at the tumor’s margin can help to guide more aggressive surgical resection strategies and targeted radiation planning to reduce recurrence. Recognizing the different habitats within a single tumor could lead to more personalized, multiregional treatment plans that target the specific biology of each subpopulation.

## 6. Linking Oxygenation to Cell Signaling and Redox Biology: Molecular Signatures, Cell States, and Immune Microenvironment

Oxygenation is essential for metabolic processes and is a key regulator of cellular function. Hypoxia triggers extensive biological reprogramming in tissues by altering molecular signatures, affecting cell states, and shaping the immune microenvironment [37,91,117,135,136,137,138]. In gliomas, many of these oxygen-dependent effects are translated through redox-sensitive signaling networks that link ROS, reactive nitrogen species (RNS), reactive sulfur species (RSS), and antioxidant buffering to invasion, stemness, immune suppression, and therapy response [25,26,139].

**Figure 2:**
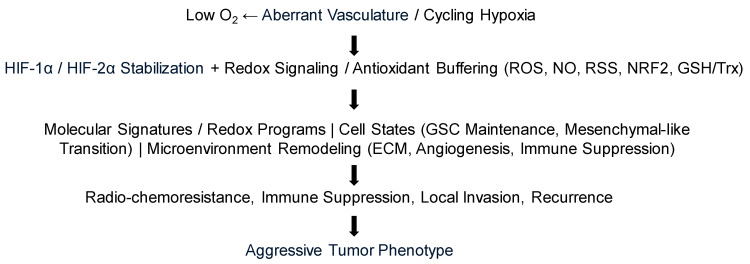
Integrative overview of how low oxygen tension and aberrant vasculature shape molecular signatures, cell states, and microenvironmental remodeling in glioblastoma through HIF stabilization and redox-sensitive signaling. The schematic summarizes how hypoxia and redox adaptation converge on metabolic rewiring, glioma stem-like cell maintenance, extracellular matrix remodeling, immune suppression, radio-chemoresistance, local invasion, and recurrence, thereby promoting an aggressive tumor phenotype. Abbreviations: HIF, hypoxia-inducible factor; ROS, reactive oxygen species; NO, nitric oxide; RSS, reactive sulfur species; NRF2, nuclear factor erythroid 2-related factor 2; GSH, glutathione; Trx, thioredoxin; GSC, glioma stem-like cell; ECM, extracellular matrix.

Stated differently, oxygenation in brain tumors is not merely a metabolic variable; it is translated into phenotype through redox-sensitive signaling nodes and post-translational modifications that determine whether oxidative stress is tolerated, buffered, or converted into cell death [27,47] (Figure 2).

### 6.1. Molecular Signatures

#### 6.1.1. Hypoxia-Inducible Factors (HIFs)

Hypoxia-driven genetic signatures are sets of genes whose expression is altered under low-oxygen conditions, and these signatures can be used to predict tumor behavior and patient outcomes [140]. The primary regulators of the cellular response to hypoxia are the HIF transcription factors, specifically HIF-1α and HIF-2α. Under normal oxygen levels (normoxia), HIF-α proteins are hydroxylated and targeted for rapid proteasomal degradation. During hypoxia, the oxygen-dependent hydroxylase enzymes are unable to function, allowing HIF-α proteins to stabilize, translocate to the nucleus, and activate the transcription of hundreds of genes. These HIF-dependent genes mediate adaptations for survival, such as increased glucose uptake, anaerobic glycolysis, and angiogenesis.

In glioma, hypoxia promotes an aggressive cellular state by upregulating genes related to migration, local invasion, immune evasion and resistance to therapy. The expression of many of these genes is directly regulated by HIFs. Key genes whose expression correlates with hypoxia in gliomas include: (i) VEGFA, which is upregulated by HIF-1 and promotes angiogenesis by stimulating new blood vessel formation; (ii) Hexokinase 2 (HK2), Lactate Dehydrogenase A (LDHA), Glyceraldehyde-3-phosphate dehydrogenase (GAPDH), and Enolase 1 or alpha-enolase (ENO1), which encode glycolytic enzymes that are often overexpressed in gliomas and are upregulated in response to hypoxia; (iii) MMP9, an enzyme that degrades extracellular matrix, promoting tumor cell invasion and migration, and is associated with recurrent, highly hypoxic gliomas; (iv) Insulin-like growth factor binding protein 2 (IGFBP2), which acts as a glioma oncogene involved in several signaling pathways and is highly expressed in hypoxic regions, particularly in GBM; (v) Lysyl oxidase (LOX), an enzyme that is highly expressed in hypoxic areas and changes the tumor microenvironment by stiffening the extracellular matrix, promoting tumor invasion. In glioma, this is more accurately framed as local invasion, mesenchymal-like transition, and recurrence than as classic systemic metastasis [134,138]; (vi) immune checkpoint pathways including programmed death-ligand 1 (PD-L1) expression and cytotoxic T-lymphocyte associated protein 4 (CTLA-4)-associated immunosuppressive signaling, which can be enhanced in hypoxic microenvironments and facilitate evasion of immune surveillance [7,37,141,142].

Hypoxia also upregulates genes involved in invasion, such as urokinase-type plasminogen activator and its receptor and contributes to epithelial-to-mesenchymal transition (EMT)-like changes, which increases the ability of cancer cells to migrate and invade (in GBM, many authors prefer mesenchymal-like or proneural-to-mesenchymal transition terminology rather than classical EMT terminology [134,138]). Lack of oxygen also drives the expression of genes that cause resistance to standard treatments like radiotherapy and temozolomide.

#### 6.1.2. Redox-Sensitive Signaling Pathways Beyond HIFs

While HIFs are central, they do not act alone. In GBM, redox homeostasis intersects with NRF2/KEAP1, PI3K/AKT/mTOR, Wnt/beta-catenin, Notch, NF-kappaB, and STAT3 signaling, thereby coupling oxidative state to self-renewal, mesenchymal-like transition, proliferation, and treatment resistance. This is particularly relevant in glioma stem-like cells, where antioxidant buffering and signaling plasticity are tightly linked [25,27,28]. Although these mechanisms are best established in GBM, a related signaling-redox interface is now emerging in aggressive meningiomas, where NF2/merlin, mTOR, and NOTCH3 pathways intersect with lipid metabolism and ferroptosis susceptibility [130,131,132].

#### 6.1.3. Reactive Oxygen Species (ROS)

Hypoxia can increase the production of ROS which, in turn, can cause DNA damage and lead to genomic instability. However, ROS-related genes are often upregulated in cancer cells, which protects them from ROS damage.

In GBM, ROS production is not only a consequence of stress but also an active signaling input. Mitochondria and NADPH oxidases, particularly NOX4 under cycling hypoxia, can drive HIF-1α stabilization, STAT3 activation, VEGF expression, and pro-invasive programs; hypoxia-induced ROS have also been linked to HIF-1alpha-SERPINE1 signaling in glioblastoma. ROS are therefore best viewed as dose- and compartment-dependent second messengers rather than uniformly toxic by-products [66,68,143].

### 6.2. Redox Homeostasis and Antioxidant Buffering

At the cellular level, oxygenation determines more than HIF stabilization; it also helps to define the redox window in which glioma cells survive. Chronic hypoxia, intermittent hypoxia–reoxygenation, and treatment-induced oxidative stress can all increase ROS formation, but the biologic outcome depends on buffering capacity rather than on ROS abundance alone. At moderate levels, ROS act as signaling mediators that support proliferation, invasion, angiogenesis, and adaptive stress responses; once buffering capacity is exceeded, oxidative injury promotes DNA damage, lipid peroxidation, and cell death.

To maintain this permissive redox window, GBM cells, and especially glioma stem-like cells, rely heavily on the glutathione and thioredoxin systems together with antioxidant enzymes and NRF2-linked stress responses. These antioxidant networks are therefore not simply background defenses; they are active determinants of stemness, invasion, radio-chemoresistance, and redox-resistant phenotypes [25,91,144].

### 6.3. Reactive Nitrogen and Sulfur Species Signaling

To better align oxygenation biology with a redox-signaling framework, it is important to extend the discussion beyond ROS to RNS and RSS. Nitric oxide (NO) can influence vascular tone, endothelial permeability, mitochondrial respiration, and immune signaling, while S-nitrosylation provides a specific covalent mechanism by which redox state alters protein function. In GBM, NO-dependent S-nitrosylation has been linked to endothelial dysfunction and vascular leakiness, and invasive GBM cells appear to exploit the transsulfuration pathway to generate cysteine for antioxidant buffering. Emerging work further suggests that hydrogen sulfide and related sulfur signaling can modulate invasion, stemness, and macrophage recruitment within the tumor microenvironment [26,121,139].

### 6.4. Redox-Dependent Post-Translational Modifications

A cell-signaling perspective also requires attention to reversible redox modifications of proteins. Oxidation of reactive cysteines, S-nitrosylation, disulfide exchange, and potentially persulfidation can alter enzyme activity, receptor trafficking, cytoskeletal organization, and endothelial barrier function. In GBM, S-nitrosylation-linked remodeling of endothelial junctional proteins provides a direct example of how redox chemistry translates into a tumor microenvironment phenotype [26,83].

### 6.5. Epigenetic Modifications

Oxygen-sensing enzymes influence epigenetic markers, including histone and DNA methylation. Hypoxia leads to significant changes in chromatin structure and gene expression patterns, further driving cellular adaptation. Many of these chromatin changes are coupled to cellular reducing power and metabolite availability, linking oxygenation, redox homeostasis, and epigenetic plasticity as integrated rather than parallel phenomena [137,145].

### 6.6. Hypoxia-Related Gene Expression Signatures Correlate with Imaging-Defined Hypoxia

In gliomas, gene expression signatures strongly correlate with imaging-defined hypoxia, particularly in aggressive, high-grade tumors like GBM, indicating that genetic signatures can serve as predictive surrogates for imaging markers [146]. Imaging techniques like PET and MRI are used to detect hypoxic regions, while genetic signatures include hypoxia-responsive genes that drive malignant behavior, treatment resistance, and poor patient prognosis [142].

Combining different types of molecular data, such as genomics, transcriptomics, and imaging, helps to create a more comprehensive understanding of a tumor’s complexity. By comparing gene expression from microdissected tumor regions with anatomical imaging, researchers have confirmed that gene signatures associated with hypoxia—such as high expression of IGFBP2 and LOX—are predominantly found in the perinecrotic and pseudopalisading zones, which are known to be highly hypoxic. A higher hypoxic risk score based on gene signatures is linked with a poorer prognosis and more aggressive tumor behavior, which aligns with findings from PET imaging showing that high [^18^F]-FMISO uptake predicts shorter survival in GBM patients. Recent spatial transcriptomic work further suggests that hypoxia acts as a long-range organizer of GBM cellular states, rather than a phenomenon confined only to the immediate perinecrotic rim [138].

The correlation between gene signatures and imaging allows for the identification of patients who may benefit from hypoxia-targeting therapies. Combining genetic and imaging information helps to better stratify patients and personalize treatment. For instance, a high-risk gene signature combined with evidence of extensive hypoxia on PET imaging may suggest the need for more aggressive treatment or therapies designed to overcome resistance.

A useful next step will be to integrate imaging-defined hypoxia with redox state classifiers such as antioxidant network signatures, transsulfuration genes, and ferroptosis regulators, rather than assuming that one hypoxia metric fully captures oxidative vulnerability [91,121,147].

### 6.7. Cell States

The molecular shifts induced by oxygenation levels influence fundamental processes in cell behavior such as differentiation, survival, and proliferation.

#### 6.7.1. Cell Differentiation

Hypoxia plays a critical role in supporting the undifferentiated state (‘stemness’) of stem and progenitor cell (glioma stem-like cell (GSC)) phenotypes, associated with self-renewal, invasion, and metabolic rewiring, which drives tumor aggressiveness and therapeutic resistance [148]. In contrast, well-oxygenated conditions promote GSC differentiation. This metabolic reprogramming is linked to increased cell migration, contributing to poor patient survival. Hypoxic GSCs are more resistant to apoptosis, chemotherapy and radiation therapy.

Redox buffering is central to this stem-like state. GSC maintenance is supported by glutathione/thioredoxin systems and by redox-linked signaling pathways including NRF2/KEAP1, AKT/c-Myc, and Wnt/beta-catenin, whereas overwhelming oxidative stress can push these cells toward differentiation or death [25,27].

#### 6.7.2. Cell Metabolism

Under low oxygen, tumor cells shift metabolism towards glycolysis, even in the presence of oxygen, a phenomenon known as the Warburg effect [145]. This metabolic shift affects cell growth, invasion, and survival, providing energy and nutrients for GSCs to survive and invade the surrounding healthy tissue. Glycolysis dominance is linked to a highly migratory state (“go”), while the pentose phosphate pathway is more associated with a proliferative state (“grow”). This shift can also lead to reprogramming of lipid metabolism, including increased triglyceride synthesis, which could potentially be exploited as a therapeutic target.

This metabolic rewiring is also a redox adaptation. Pathways such as the pentose phosphate pathway, serine synthesis, one-carbon metabolism, and transsulfuration help generate NADPH and cysteine to sustain glutathione and thioredoxin systems under hypoxia, nutrient limitation, and therapy-induced oxidative stress. Thus, metabolic plasticity and redox buffering are mechanistically inseparable in GBM [121,145,149].

#### 6.7.3. Redox-Dependent Cell Death Programs

Redox imbalance also influences which cell death program dominates in brain tumors. While severe oxidative injury can contribute to apoptosis and necrotic damage, a major current focus is ferroptosis, an iron-dependent form of cell death driven by lipid peroxidation. In GBM, ferroptosis sensitivity is shaped by SLC7A11/xCT activity, glutathione availability, GPX4-linked protection, and NRF2 status, creating a potential therapeutic opportunity in tumors that resist conventional apoptosis [144,150,151].

Parallel concepts are beginning to emerge in malignant meningioma. NF2 loss and low E-cadherin can sensitize meningioma cells to erastin-induced ferroptosis, NOTCH3 can promote ferroptosis resistance through fatty acid oxidation, and mifepristone has recently been reported to induce ferroptosis through a PR/p53/HO1/GPX4 axis, suggesting that ferroptosis-oriented strategies may extend beyond glioma into selected non-glial brain tumors [131,132,152].

### 6.8. Immune Microenvironment

Oxygen gradients within tissues are crucial for regulating immune cell function and shaping the overall immune microenvironment. In chronic inflammation and cancer, hypoxia can create an immunosuppressive microenvironment, promoting the accumulation of immunosuppressive cells, which inhibit anti-tumor immune responses, and directly reducing the effectiveness of immunotherapies. Conversely, adequate oxygenation may enhance immune cell activation and improve the efficacy of immunotherapies.

#### Effects of Hypoxia on Immune Response

Hypoxia reduces cytokine production and T cell proliferation and infiltration, leading to T cell exhaustion and decreased cytotoxicity [37]. It also promotes T cell apoptosis and can make tumor cells resistant to T cell-mediated killing, even if the T cells are not directly impaired in vitro. The accumulation and activation of immunosuppressive myeloid cells, such as MDSCs and M2 macrophages, further dampen anti-tumor immunity. Low oxygenation also impairs the maturation and function of antigen-presenting cells like dendritic cells, which are crucial for starting an immune response. Other immune cells, such as natural killer cells and gamma delta T cells (γδ T cells), are also negatively affected by hypoxia, leading to attenuated or suppressed anti-tumor activity. Recent spatial studies in GBM indicate that hypoxia helps to organize immunosuppressive myeloid states and can promote macrophage/microglial polarization toward tumor-supportive phenotypes [117,153].

The combined effect of suppressed effector cells and activated suppressive cells creates a highly immune-suppressed microenvironment that limits the success of T cell-based therapies such as immune checkpoint inhibitors. Hypoxia also induces expression of immune checkpoint molecules like PD-L1 and CTLA-4 on tumor cells and immune cells, creating a physical and functional barrier to T cell activation. These tumors are thus inherently immunologically “cold” (i.e., lack immune function), making them poor candidates for these therapies. Overcoming hypoxia through strategies like supplemental oxygen, oxygen-supplied nanomaterials or by combining immunotherapy with other treatments that normalize tumor vasculature to improve outcomes is a promising area of research [37].

From a redox perspective, immune suppression in GBM is not only a consequence of low oxygen but also of oxidative signaling. ROS, NO, lipid peroxidation products, and sulfur-metabolic cues can influence macrophage polarization, T cell dysfunction, endothelial activation, and myeloid cell recruitment, helping to explain why hypoxic and redox-buffered tumors often behave as immunologically cold lesions [117,139,153].

The link between oxygenation and biological outcomes is complex and context dependent. The interplay among molecular signatures, cell states, and the immune microenvironment highlights how oxygen levels function as a fundamental regulator of physiology and disease. Researchers are using this understanding to develop therapeutic strategies that target oxygen-sensing pathways in conditions like cancer, inflammatory disorders, and tissue regeneration.

### 6.9. Redox-Targeted Therapeutic Applications

Because oxygenation changes are ultimately translated through redox-sensitive pathways, it is useful to distinguish oxygen-modifying strategies from redox-targeted strategies. Oxygen delivery approaches such as hyperbaric oxygen, carbogen, and vascular normalization attempt to alter the upstream microenvironment, whereas redox-targeted strategies aim to push tumor cells outside their tolerated oxidative range. In GBM, relevant examples include inhibition of thioredoxin reductase, dual disruption of glutathione and thioredoxin buffering, targeting cysteine/transsulfuration metabolism, and ferroptosis-oriented approaches that exploit glutathione dependence. Even where clinical translation remains early, this framework better links molecular mechanism to therapeutic application and aligns hypoxia imaging with biomarker-guided precision therapy [25,144,154].

In practical terms, redox-directed therapy can be grouped into three strategies: disabling antioxidant defenses, forcing toxic ROS/lipid peroxidation, or exploiting tumors that are already near their oxidative threshold. Candidate approaches in GBM therefore include thioredoxin reductase inhibition, glutathione depletion, xCT/SLC7A11 targeting, transsulfuration inhibition, ferroptosis induction, and selected ROS-generating local therapies. The major translational challenge is matching the right oxidative intervention to the right tumor state [25,122,150,154].

## 7. Clinical Implications and Oxygen- and Redox-Targeted Strategies in Neuro-Oncology

### 7.1. Limitations of Models Relevant to Tissue Oxygenation and Redox States

In brain slice preparations kept under controlled perfusion in vitro, the deeper layers are often functionally compromised or necrotic because diffusion of oxygen and nutrients is insufficient to reach the tissue core [11,39,48,155,156,157]. This creates a diffusion-limited geometry that differs substantially from in vivo perfused tumor tissue. In vivo, oxygen is supplied efficiently via a dense capillary network that ensures all cells receive adequate perfusion. In vitro slices rely solely on surface diffusion, which does not replicate the natural physiological oxygen gradient and supply dynamics. Artificial cerebrospinal fluid (aCSF) without oxygen carriers like red blood cells, only provides effective oxygenation to a depth of a few hundred micrometers, thus the core of thicker slices is hypoxic. As neurons have a high metabolic rate, available oxygen is rapidly consumed, increasing hypoxia in the slice core. These limitations mean that ex vivo brain slices, while invaluable mechanistic models for research in synaptic plasticity or network activity where superficial layers are the primary focus, are imperfect surrogates for in vivo oxygen dynamics [39,155,156,157].

**Figure 3:**
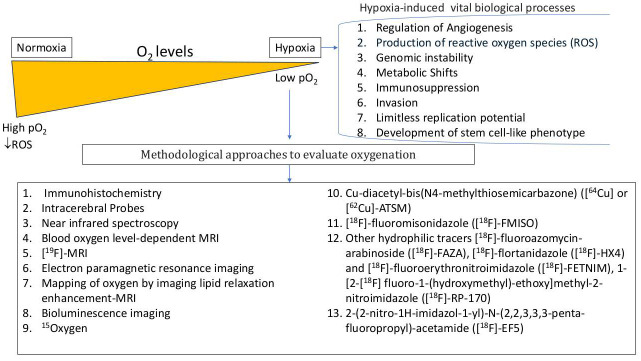
Relationship between oxygen gradients, hypoxia-associated biologic processes, and methods used to evaluate tumor oxygenation. The schematic summarizes how declining oxygen levels in brain tumors are linked to angiogenesis, ROS production, genomic instability, metabolic shifts, immunosuppression, invasion, and stem-like phenotypes, alongside representative invasive and imaging-based approaches used to assess oxygenation.

Approaches to modify oxygenation in brain tumors have focused on enhancing the efficacy of conventional therapies like radiation and chemotherapy, which are often hindered by the tumor’s hypoxic microenvironment (Figure 3).

### 7.2. Direct Redox Modulation

Beyond manipulating oxygen delivery, an equally important therapeutic concept is direct modulation of the tumor redox state. This includes disabling glutathione or thioredoxin buffering, targeting cysteine supply, and sensitizing tumor cells to ferroptosis or other oxidative death programs. Such approaches may be especially relevant when imaging suggests persistent hypoxia but molecular profiling suggests limited antioxidant reserve, because oxygen-normalizing interventions alone may not reverse downstream redox adaptations [25,144,154].

Although still preclinical, a similar logic may be relevant in recurrent or high-grade meningioma, where oxidative stress-based classification, mTOR/redox coupling, and ferroptosis sensitivity have begun to define potentially actionable vulnerabilities [129,130,152].

### 7.3. Hyperbaric Oxygen Therapy (HBOT)

HBOT involves breathing 100% oxygen at pressures greater than one standard atmosphere, which significantly increases the amount of oxygen dissolved in the plasma and delivered to tissues, including tumors [158,159,160,161]. Early clinical trials in patients with high-grade gliomas showed promising results when HBOT was combined with radiotherapy, suggesting a survival benefit. Preclinical studies in human glioma models (U87) also confirmed that HBOT improved tumor oxygenation and enhanced the effects of certain chemotherapy drugs. Despite positive outcomes in some studies, the complexity of the procedure, patient compliance issues, and logistical challenges led to the approach being dropped for general cancer treatment. However, recent research suggests that HBOT can reduce radiation-induced brain injury and may improve outcomes when combined with modern therapies, prompting calls for further prospective randomized studies.

### 7.4. Carbogen Breathing

When patients inhale carbogen, a gas mixture of 95% oxygen and 5% carbon dioxide [162], the high oxygen content increases dissolved oxygen, while the carbon dioxide acts as a vasodilator, further enhancing blood flow and oxygen delivery to the tumor. Carbogen breathing can increase arterial oxygenation in patients with GBM. However, the efficacy is highly dependent on the capacity of the individual tumor vascular system to dilate, which is often irregular and poor in highly aggressive GBMs, leading to inconsistent results. Clinical trials combining carbogen with radiotherapy (e.g., the ARCON protocol) were started, but early results showed high toxicity (e.g., acute liver toxicity and potential CNS toxicity) and limited clinical benefit for malignant gliomas.

### 7.5. Anti-Angiogenic “Normalization”

This strategy uses anti-angiogenic agents (which block the formation of new blood vessels) to prune the abnormal, leaky tumor vasculature into a more normal, functional state [163]. This improves blood flow and oxygen/drug delivery to the tumor, a concept known as “vascular normalization”. GBMs are highly vascularized, making them a prime target for this approach. Agents like bevacizumab (Avastin), an anti-VEGF-A antibody, have been widely studied. They provide potent anti-edema effects, reducing brain swelling and allowing for a reduction in steroid use. However, despite promising phase II results showing improved progression-free survival and high radiographic response rates, large phase III clinical trials for newly diagnosed or recurrent GBM have not demonstrated an improvement in overall survival. As resistance inevitably develops through alternative pro-angiogenic pathways, this approach is primarily used for symptom management (edema control) and improving quality of life, rather than as a curative strategy. The putative normalization window is also variable and not universal. Importantly, phase III trials of bevacizumab in newly diagnosed GBM improved progression-free survival but not overall survival, so oxygenation gains should be viewed as biologically attractive but clinically unsettled [164,165].

### 7.6. Hypoxia-Activated Prodrugs (HAPs)

HAPs are inactive compounds that are selectively converted into active, cytotoxic drugs under the low-oxygen conditions found in solid tumors [166], thereby targeting only the hypoxic cancer cells while sparing normal, oxygenated tissue. Gliomas show significant hypoxic microenvironments, making them theoretically attractive targets for HAPs, and specific agents like tirapazamine and evofosfamide (TH-302) have been evaluated. Tirapazamine showed encouraging preclinical results, but showed inconsistent efficacy and notable toxicities (muscle cramping, ototoxicity) in clinical trials. Evofosfamide (TH-302), which can penetrate the blood–brain barrier, was investigated in phase I/II trials for GBM. However, phase III clinical trials for various cancers, including specific results not detailed for GBM, failed, partly due to patient selection based on actual tumor hypoxia status. HAPs have shown great potential in preclinical studies, but their clinical translation in brain tumors has been disappointing due to challenges like overcoming the blood–brain barrier and the need for reliable imaging biomarkers to identify suitable patients. Current research focuses on improving drug design and combining HAPs with other modalities, such as nanomedicine [167].

ROS-generating locoregional approaches such as photodynamic therapy and sonodynamic therapy are also increasingly relevant to a redox-oriented framework. Their biologic appeal lies in the localized generation of cytotoxic ROS, vascular effects, and potential immune modulation; in brain tumors, these strategies are advancing clinically but still depend on adequate sensitizer delivery and careful consideration of hypoxic constraints [168,169].

### 7.7. Oxygenation Mapping

Imaging can be integrated into cancer treatment to find and map hypoxic tumor regions, which are often resistant to standard therapies. This information is used to personalize and adapt radiotherapy, guide surgical approaches, and stratify patients or monitor response in systemic therapy trials. Characterizing altered responses to controlled CO_2_ and O_2_ respiratory challenges with BOLD-fMRI provides important information for neurosurgical planning. It helps to identify true functional tissue from compromised tissue, predict tumor boundaries beyond structural images, and assess therapeutic response. The dynamic and often contradictory effects of both radiotherapy and anti-angiogenic therapy on tumor oxygenation highlight the need for personalized and carefully timed treatment strategies. Monitoring tumor oxygenation levels throughout treatment with advanced imaging techniques could help to identify the best “window” for combination therapy to maximize effectiveness.

### 7.8. Radiotherapy Planning

Hypoxia is a major cause of radioresistance, because oxygen is required for radiation to effectively kill cancer cells [32]. Oxygenation imaging techniques like PET (using tracers such as ^18^F-FMISO) or MRI (using BOLD or TOLD methods) are used to create “biological target volumes”. Higher radiation doses are then delivered specifically to these radioresistant hypoxic regions to achieve better local tumor control, while sparing surrounding healthy tissue. Repeated imaging during treatment can track changes in oxygenation (reoxygenation) as the tumor shrinks. Treatment plans can be adjusted dynamically to target persistent hypoxic areas or to time radiation delivery with peak oxygenation. Identifying patients with highly hypoxic tumors before treatment helps clinicians to select the best strategy, such as combining radiation with novel oxygen-modifying agents or radiosensitizers.

### 7.9. Surgery

Oxygenation imaging is not yet standard for extent-of-resection decisions in neuro-oncology, but it may still provide clinically relevant physiologic context. Potential applications include identifying infiltrative peritumoral zones that are more likely to harbor treatment-resistant disease, distinguishing eloquent but physiologically compromised cortex from more preserved tissue, and improving interpretation of preoperative functional mapping in regions affected by neurovascular uncoupling [73]. Accordingly, the most relevant surgical role in brain tumors is not extrapolation from lymph-node or superficial-cancer workflows, but improved interpretation of tumor margins, peritumoral tissue, and functional risk in individual patients.

### 7.10. Systemic Therapy Trials

In clinical studies specifically related to hypoxia assessment or oxygen-modifying therapy in GBM (Table 3), oxygenation imaging serves as a vital biomarker, predicting response and prognosis. As hypoxia is a known negative prognostic indicator for many cancer treatments, including chemotherapy and targeted therapies [37], imaging baseline oxygenation levels helps to predict patient outcomes. This information can also be used to stratify patients into different treatment arms within clinical trials. Oxygenation imaging can be used as an early indicator of treatment efficacy; a significant increase in tumor oxygenation following a new therapy may correlate with a positive outcome, even before changes in tumor size are detectable. Clinical trials are actively using oxygen-enhanced MRI and PET to evaluate and confirm the effectiveness of hypoxia-modifying drugs or to identify patients likely to respond well to immunotherapy. This allows researchers to better understand the mechanisms of action for new therapies. A redox-aware trial design may be particularly useful here: oxygenation imaging could be combined with tumor markers of antioxidant capacity, transsulfuration, NRF2 activation, or ferroptosis susceptibility to determine patients most likely to benefit from redox-modulating therapy [91,121,144].

### 7.11. Artificial Intelligence (AI)

AI, particularly machine learning and deep learning, acts as an integrating force. Algorithms can process and combine vast, disparate datasets from medical images (radiomics features), genomic profiles, and clinical information, including oxygenation metrics [147,170], and automate the labor-intensive process of segmenting tumors and extracting subtle, complex features from images that may be imperceptible to the human eye. Based on the hypothesis that genetic and molecular alterations in a tumor manifest as detectable patterns in medical images, AI builds robust predictive models to stratify patients, based on their integrated data, to predict treatment response (e.g., to anti-angiogenic therapy or radiotherapy) and overall prognosis and survival.

Radiogenomics—an emerging area of precision medicine—combines oxygenation metrics with the presence of specific genetic mutations or pathways associated with hypoxia, and uses AI to provide a comprehensive, non-invasive approach to patient stratification and the development of personalized cancer treatment strategies. By finding these correlations, researchers can develop non-invasive imaging biomarkers that reflect the tumor’s genetic profile and biological behavior, including its hypoxic status.

AI and radiogenomics may ultimately help to connect macro-scale oxygen maps with micro-scale redox biology by integrating FMISO/OE-MRI features with antioxidant network signatures, ferroptosis regulators, and immune cell states. This type of multiscale model would better fit the biologic reality that oxygenation, signaling, and redox homeostasis co-evolve rather than acting independently [9,91,147].

## 8. Conclusions

In brain tumors, compromised oxygenation results from a destructive interplay between the competing high metabolic needs of the tumor and those of surrounding neurons, and the mass effects of edema. This leads to a hypoxic, acidic, immunologically cold microenvironment that promotes tumor aggression, and is a major obstacle to effective treatment. Clinical strategies to image hypoxic regions and modulate oxygenation are promising, but remain limited in routine practice. Future progress will require noninvasive tools that can be integrated into clinical workflows and interpreted with explicit attention to what each modality does, and does not, measure. Targeting hypoxia-induced pathways in combination with immunotherapy remains promising, but stronger biomarker-guided clinical validation is needed.

Clinical strategies that directly improve or exploit tumor oxygenation remain limited, underscoring the need for better tools to identify actionable hypoxic niches over time. Targeting hypoxia-induced pathways together with redox-sensitive vulnerabilities, radiotherapy, and immunotherapy may offer a more rational precision medicine framework for neuro-oncology. Taken together, glioma oxygenation should be framed not only as a perfusion and imaging problem but also as a redox signaling problem. This perspective integrates oxygen gradients with ROS, RNS, sulfur-linked metabolism, antioxidant buffering, cell state plasticity, and therapy response [25,91,139]. Hypoxia-directed imaging may therefore be most informative when paired with redox-aware biomarkers and therapeutic strategies.

## Figures and Tables

**Table 1:** Common imaging modalities used before and after tumor resection in GBM Patients.

Phase	Imaging Type	Common Modalities/Tracers Used	Purpose
Pre-Surgery	Convention a l MRI	T1-CE (Gadolinium), T2/FLAIR	Define tumor anatomy, necrosis, peritumoral edema, and enhancement patterns.
Pre-Surgery	Advanced MRI	DSC/PWI (Perfusion), DWI (Diffusion), MRS (Spectroscopy), fMRI/DTI	Assess tumor vascularity (CBV), cellularity, metabolic ratios (Cho/NAA), and identify eloquent areas for surgical planning.
Pre-Surgery	PET (Preferred)	^11^C-MET (Methionine), ^18^F-FET (Fluoro-ethyl-tyrosine), ^18^F-FDOPA	Delineate true tumor boundaries, identify tumor infiltration beyond T2-FLAIR signal, and guide biopsy.
Surgery	Intraoperative	iMRI (0.5–3 T), Fluorescence (5-ALA, Fluorescein)	Maximize the extent of resection (EOR) and account for brain shift in real-time.
Post-Surgery	Early Post-op MRI	T1-CE (Within 24–72 h)	Baseline scan: Evaluate residual enhancement, surgical complications (hemorrhage, infarcts), and guide radiation.
Post-Surgery	Surveillance/Recurrence	Advanced MRI (DSC, MRS), AminoAcid PET (^11^C-MET, ^18^F-FET)	Differentiate true tumor recurrence from pseudo-progression (treatment effect/radiation necrosis).

Abbreviations: CBV: Cerebral blood volume; Cho/NAA: Choline/N-acetylaspartate (Cho/NAA) ratio; DWI: diffusion-weighted imaging; DTI: Diffusion tensor imaging; DSCPWI: Dynamic susceptibility contrast perfusion-weighted imaging; EOR: extent of resection; EPMR: Early Postoperative MRI; fMRI: functional MRI; iMRI: intraoperative MRI; MRI: magnetic resonance imaging; MRS: MR Spectroscopy; PET: positron emission tomography; T1-CE: T1-weighted with Gadolinium;.

**Table 2:** Summary of techniques used to assess oxygenation in brain tumors.

Technique	Principal Readout	Key Strengths	Key limitations/Interpretation
Polarographic oxygen electrodes	Local tissue pO_2_	Direct quantitative real-time point measurement; commonly treated as a reference standard	Invasive, highly focal, location-dependent, and impractical for serial sampling
Fiber-optic probes	Focal oxygen-dependent luminescence/local pO_2_	Sensitive at low O_2_ and can provide real-time focal measurements	Invasive and spatially limited; poor whole-tumor representation
EPR oximetry	Local tissue pO_2_ at implanted sites	Allows serial and multi-site measurements without repeated probe insertion	Requires implanted probes and has limited clinical availability
BOLD/T2* MRI	Deoxyhemoglobin-sensitive signal and vascular responsiveness	Non-invasive, widely available, high spatiotemporal resolution	Indirect and relative; influenced by blood flow, blood volume, and neurovascular uncoupling
qBOLD	OEF/DBV and estimated CMRO_2_	Model-based quantitative oxygen-metabolism estimates	Dependent on biophysical assumptions and susceptible to noise/model error
Perfusion MRI (DSC/DCE/ASL)	Blood delivery and microvascular perfusion	Clinically accessible with good spatial detail and useful adjunctive vascular information	Does not directly measure tissue pO_2_
OE-MRI/TOLD	Signal change during oxygen challenge	Repeatable mapping of oxygen responsiveness	Semiquantitative; should not be interpreted as a universal absolute pO_2_ map
Nitroimidazole PET (e.g., FMISO)	Tracer retention in hypoxic tissue	Whole-tumor mapping with molecular specificity for severe hypoxia	Radiation exposure, lower spatial resolution, delayed imaging, and threshold dependence

**Table 3:** Representative clinical studies and trials relevant to hypoxia assessment or oxygen-modifying strategies in GBM.

Identifier	Approach/Study Focus	Relevance to Hypoxia or Oxygenation
NCT00430079	EF5-based assessment of hypoxia in malignant glioma	Early clinical effort to characterize tumor hypoxia directly in malignant glioma
NCT00906893	[^18^F]-FMISO PET in non-operated glioblastoma	Pre-treatment PET mapping of hypoxic tumor regions
NCT00902577	ACRIN 6684: FMISO PET and MRI in newly diagnosed GBM	Multimodal imaging study of hypoxia before radiotherapy
NCT02466828	qBOLD MRI of GBM for assessment of tumor hypoxia	MRI-based quantification of oxygen-related parameters
NCT02076152	FMISO-PET and MRI in GBM	Imaging study linking hypoxia assessment to therapeutic response
NCT03573986	FMISO PET/CT and MRI before and after bevacizumab in recurrent GBM	Tracks vascular and hypoxia-related changes with anti-angiogenic therapy
NCT03216499	HIF-2α inhibitor PT2385 in recurrent glioblastoma	Direct targeting of hypoxia signaling rather than oxygen delivery alone
NCT03862430	NanO_2_ combined with radiation and temozolomide	Representative oxygen-modifying therapeutic strategy
NCT05500612	OE-MRI/BOLD MRI hypoxia study for GBM radiotherapy	MRI-based identification of hypoxic tumor habitats for treatment planning
NCT06477939	Liposomal transcrocetin with hypofractionated radiotherapy and temozolomide	Oxygen-delivery strategy intended to counter tumor hypoxia
NCT07417774	Liquid biopsy substudy in GBM treated with chemoradiation and an oxygen therapeutic	Biomarker-focused study paired with an oxygen-modifying therapy

## Data Availability

No new data were created or analyzed in this study. Data sharing is not applicable to this article.

## Abbreviations

The following abbreviations are used in this manuscript:

aCSF	Artificial cerebrospinal fluid
AI	Artificial intelligence
BOLD	Blood oxygen level-dependent
CNS	Central nervous system
CMRO_2_	Cerebral metabolic rate of oxygen
CPP	Cerebral perfusion pressure
CT	Computed tomography
CTLA-4	Cytotoxic T-lymphocyte associated protein 4
DBV	Deoxygenated blood volume
DCE (MRI)	Dynamic contrast enhanced (MRI)
DNA	Deoxyribonucleic acid
DSC (MRI)	Dynamic susceptibility contrast (MRI)
EMT	Epithelial-to-mesenchymal transition
ENO1	Enolase 1 or alpha-enolase
EPR	Electron paramagnetic resonance
FLAIR	Fluid-attenuated inversion recovery
FMISO	^18^F Fluoromisonidazole
fMRI	Functional magnetic resonance imaging
fNIRS	functional near-infrared spectroscopy
GAPDH	Glyceraldehyde-3-phosphate dehydrogenase
GBM	Glioblastoma
GSC	Glioma stem-like cell
HAP	Hypoxia-activated prodrug
HBOT	Hyperbaric oxygen therapy
HGG	High-grade glioma
HK2	Hexokinase 2
HIF	Hypoxia-inducible factor
ICP	Intracranial pressure
IDH	Isocitrate dehydrogenase
IGBP2	Insulin-like growth factor binding protein 2
LDHA	Lactate dehydrogenase A
LGG	Low-grade glioma
LOX	Lysyl oxidase
MRI	Magnetic resonance imaging
NADPH	Nicotinamide adenine dinucleotide phosphate
NO	Nitric oxide
NRF2	Nuclear factor erythroid 2-related factor 2
NVC	Neurovascular coupling
OEF	Oxygen extraction fraction
OE-MRI	Oxygen-enhanced MRI
PAI	Photoacoustic imaging
PALT	Photoacoustic lifetime
PD-L1	Programmed death-ligand-1
PET	Positron emission tomography
pO_2_	Partial pressure of oxygen
PBZ	Peritumoral brain zone
qBOLD	Quantitative BOLD
RNA	Ribonucleic acid
ROS	Reactive oxygen species
RNS	Reactive nitrogen species
RSS	Reactive sulfur species
SBRT	Stereotactic body radiotherapy
TOLD	Tissue oxygen level-dependent (MRI)
VEGF	Vascular endothelial growth factor
